# Mulberry leaves extract ameliorates alcohol-induced liver damages through reduction of acetaldehyde toxicity and inhibition of apoptosis caused by oxidative stress signals

**DOI:** 10.7150/ijms.50174

**Published:** 2021-01-01

**Authors:** Hsin-Wen Liang, Tsung-Yuan Yang, Chia-Sheng Teng, Yi-Ju Lee, Meng-Hsun Yu, Huei-Jane Lee, Li-Sung Hsu, Chau-Jong Wang

**Affiliations:** 1 Institute of Biochemistry, Microbiology, and Immunology, Chung Shan Medical University, Taichung, 402, Taiwan; 2 Department of Internal Medicine, Chung-Shan Medical University Hospital, Taichung 402, Taiwan; 3 School of Medicine, Institute of Medicine, Chung Shan Medical University, Taichung 402, Taiwan; 4 Department of Pathology, School of Medicine, Chung Shan Medical University, Taichung City 402, Taiwan; 5 Department of Pathology, Chung Shan Medical University Hospital, Taichung City 402, Taiwan; 6 Department of Biochemistry, School of Medicine, Medical College, Chung Shan Medical University, Taichung, 402, Taiwan; 7 Clinical Laboratory, Chung Shan Medical University Hospital, Taichung, 402, Taiwan; 8 Department of Health Diet and Industry Management, Chung Shan Medical University, Taichung 402, Taiwan; 9 Department of Medical Research, Chung Shan Medical University Hospital, Taichung, 402, Taiwan

**Keywords:** alcohol liver disease, mulberry leaf extract, oxidative stress, inflammation, acetaldehyde, apoptosis

## Abstract

Mulberry leaves (Morus alba L.), which are traditional Chinese herbs, exert several biological functions, such as antioxidant, anti-inflammation, antidiabetic, and antitumor. Alcohol intake increases inflammation and oxidative stress, and this increase causes liver injury and leads to liver steatosis, cirrhosis, and hepatocellular carcinoma, which are major health problems worldwide. Previous report indicated that mulberry leaf extract (MLE) exited hepatoprotection effects against chronic alcohol-induced liver damages. In this present study, we investigated the effects of MLE on acute alcohol and liver injury induced by its metabolized compound called acetaldehyde (ACE) by using in vivo and in vitro models. Administration of MLE reversed acute alcohol-induced liver damages, increased acetaldehyde (ACE) level, and decreased aldehyde dehydrogenase activity in a dose-dependent manner. Acute alcohol exposure-induced leukocyte infiltration and pro-inflammation factors, including cyclooxygenase-2 (COX-2), tumor necrosis factor-α (TNF-α), and interleukin-6 (IL-6), were blocked by MLE in proportion to MLE concentration. MLE prevented alcohol-induced liver apoptosis via enhanced caveolin-1 expression and attenuated EGFR/STAT3/iNOS pathway using immunohistochemical analysis. ACE induced proteins, such as iNOS, COX-2, TNF-α, and IL-6, and inhibited superoxide dismutase expression, whereas co-treated with MLE reversed these proteins expression. MLE also recovered alcohol-induced apoptosis in cultured Hep G2 cells. Overall, our findings indicated that MLE ameliorated acute alcohol-induced liver damages by reducing ACE toxicity and inhibiting apoptosis caused by oxidative stress signals. Our results implied that MLE might be a potential agent for treating alcohol liver disease.

## Introduction

Alcohol liver disease (ALD), which leads to hepatic steatosis, cirrhosis, and hepatocellular carcinoma due to alcohol consumption, is a critical health problem all over the world 1. Alcohol is converted into acetaldehyde (ACE), which is a harmful substance, by alcohol dehydrogenase (ADH) in liver 2. ACE is subsequently metabolized into acetate in liver by aldehyde dehydrogenase (ALDH) 2. During the alcohol metabolism processes, free radicals, such as reactive oxygen species (ROS) and reactive nitrogen species (RNS), are generated 3, 4. ROS and RNS play a critical role in cell apoptosis, inflammation, and fibrosis 4. Several endogenous antioxidant enzymes, such as catalase (CAT), superoxide dismutase (SOD), and glutathione peroxidase (GSH-Px), have been generated to maintain homeostasis of oxidative stress 5. Reports have shown that chronic exposure to alcohol influences the levels of these antioxidant enzymes, increases the levels of ROS, and causes injury to the liver 6. Alcohol exposure may enhance the nitride oxide (NO) level 3. Depletion of inducible NO synthase (iNOS) significantly attenuates alcohol-induced liver damages 7. Another risk factor for alcohol-induced liver damages is inflammation. Accumulated ROS elevates the damage-associated molecular patterns, triggers the pro-inflammation cytokines, such as tumor necrosis factor-α (TNF-α) and interleukin-1 (IL-1), and contributes to the progression of ALD 8.

Mulberry (*Morus alba* L.) is a native plant that is mostly cultured in China, Korea, and Japan 9. Mulberry exhibits several cellular functions, such as anti-atherosclerosis 10, anti-diabetic 11, and liver protection 9, 12. Mulberry leaves polyphenols attenuated lipid accumulation in liver through downregulation of fatty acid synthase and acetyl-CoA carboxylase and promoted AMP-dependent protein kinase pathway 13. Mulberry methanol extracts (MME) significantly attenuate high fat diet (HFD)-induced body weight increase, liver lipid droplet accumulation, and liver steatosis 14. MME also reverses the expression of HFD-induced glycerol kinase and fatty acid desaturase 2 14. Tang et al. demonstrated that administration of mulberry water extract (MWE) at concentrations of 1% and 2% significantly attenuates ethanol-induced liver damage markers and lipid synthesis proteins 15. MWE also increases the lipid metabolism protein expression and the activities of AMP-dependent protein kinase and peroxisome proliferator-activated receptor-α 15. Mulberry leaves extracts also prevented non-alcoholic fatty liver disease (NFALD) via enhanced anti-oxidant enzyme expression and diminished inflammatory cytokine expression 16. Previous reports have indicated that chronic exposure to alcohol for 8 weeks significantly induces liver injury biomarkers, whereas co-administration of mulberry leaf extract (MLE) recovers the injury to normal conditions 17. MLE also attenuates chronic alcohol-induced liver oxidative stress 17. MLE enhances the expression of caveolin-1 and blocks epidermal growth factor receptor (EGFR), signal transducer and activator of transcription 3 (STAT3), and iNOS pathway to recover alcohol-induced liver damages 17.

In the present study, we investigated the hepatoprotection and antioxidative stress effects of MLE on acute alcohol-induced liver damage. The liver markers, antioxidant enzyme expression, ADH activity, and inflammation cytokine expression were analyzed in ethanol-exposed mice with or without MLE cotreatment.

## Materials and methods

### MLE preparation

Dried mulberry leaves (100 g) were added into 3000 mL distilled water and boiled at 90 °C for 2 h. The supernatants (20 L) were collected, filtered using a 0.2 μM filter, and lyophilized under reduced pressure. The powders were stored at room temperature until use. A total of 100 g mulberry leaf extract produced 32 g polyphenol-rich MLE a yield of 32%). The compounds of MLE were analyzed by HPLC method as previous reported 17.

### Animals and experimental design

The ICR mice (body weight of around 30 g) were purchased from LASCO Inc (Taipei, Taiwan). All animal experimental protocols used in this study were approved by the Institutional Animal Care and Use Committee of the Chung Shan Medical University. The mice were housed and acclimated in 22 ± 2 °C with 12 h light/dark cycle. A total of 40 mice were divided into (1) control, (2) ethanol containing 5% (v/v) ethanol (40% ethanol-derived calories), (3) ethanol + 0.5% MLE, (4) ethanol + 1% MLE, and (5) ethanol + 2% MLE. For MLE groups, the MLE was administrated 0.5 h before ethanol treatment. The blood was collected 1.5 h post-ethanol treatment. The mice were sacrificed, and liver tissue was collected 3 h post-ethanol treatment.

### Detection of serum ACE concentration

The serum ACE was measured by Sigma-Aldrich Colorimetric ACE assay kit. Briefly, 25 μL serum was mixed with 75 μL dilution buffer and incubated at room temperature for 1 h in dark. The absorption was determined at 405 nm. The concentration of ACE was estimated following the protocols of the manufacturer.

### Measure of ADH and ALDH activities

For ALDH activity assay, 25 μL serum was mixed with 500 μL reaction buffer (3.6 mM ACE + 1 mM β-NAD + 20 mM pyrazole, pH 8.6). The absorption at 340 nm was detected every 60 s for 20 min. The activities were calculated according to the absorption.

### Preparation of liver homogenate

Liver tissue (0.3 g) was added to 3 mL phosphate-EDTA buffer and homogenized using a homogenizer on ice. The homogenates were obtained after being centrifuged at 3000 × g for 30 min followed by 12 000 × g for 5 min.

### Measurement of the activities of antioxidant enzymes

Ten μL liver homogenates were subjected to analyze the SOD, CAT, and GSH-Px activities as in previous reports 17, 18. CAT was measured by adding 0.03 M H2O2, and the absorption was detected at 240 nm. SOD activity was analyzed by pyrogallol autoxidation assay. GSH-Px activity was determined by the ability to convert GSH into oxidized form using H_2_O_2_.

### Immunohistochemical staining

Paraffin-embedded liver section was deparaffinized, rehydrated, and treated with 3% for 10 min. The sections were washed with phosphate-buffered saline (PBS) and then incubated with antibodies against SOD, caveolin-1, iNOS, p-STAT3, and p-EGFR at 4 °C overnight. After being washed with PBS, the sections were reacted with horseradish peroxidase-conjugated secondary antibodies. The color was developed by 3,3′-diaminobenzidine, and counterstaining with hematoxylin was conducted.

### Cell culture and treatment

Human Hep G2 cells were obtained from American Type Culture Collection and maintained in minimal essential medium supplemented with 10% fetal bovine serum, 1% glutamine, and 1% nonessential amino acids. These cells were cultured in 37 °C incubator under a humidified atmosphere with 5% CO2. For study, Hep G2 cells were treated with 50 mM ethanol or 175 μM ACE for 24 h.

### MTT assay

Hep G2 cells were seeded into 24-well plate in a density of 3 × 10^4^ and treated with 0, 1, 2, 4, 6, 8, and 10 mg/mL MLE for 24 h. The medium was replaced with fresh medium containing 0.5 mg/mL MTT (3,4,5-cimethylthiazol-2-yl-2,5-diphenyl tetrazolium bromide) and cultured for additional 4 h. The purple-blue formazan was dissolved in 1 mL of isopropanol. The cell viability was measured by the absorbance of OD563 in ethanol-treated groups compared with the vehicle groups.

### Western blot analysis

The protein concentration of liver homogenate was measured using Bio-Rad protein assay kit. Fifty mg protein was separated by SDS-polyacrylamide gel electrophoresis and then transferred onto nitrocellulose membranes. The membranes were blocked with 5% nonfat milk, incubated with specific antibodies, and probed with secondary antibodies. Positive signals were detected by enhanced chemiluminescence using ECL Western blot detection reagents. The band intensity was measured using Multi Gauge V2.2 software.

### Statistical analysis

Data were presented as mean ± SEM. Student's t test was performed to detect the significances using Sigma Plot version 10. p < 0.05 was considered a significant difference.

## Results

### Effects of MLE on ethanol metabolism and ALDH activity

MLE was extracted as previous report 17. The major compounds and their percentage of MLE were neochlorogenic acid (0.355%), cryptochlorogenic acid (0.317%), chlorogenic acid (0.238%), rutin (0.092%), isoquercitrin (0.056%), astragalin (0.053%), nicotiflorin (0.035%), and protocatechuic acid (0.013%).

**Figure 1:**
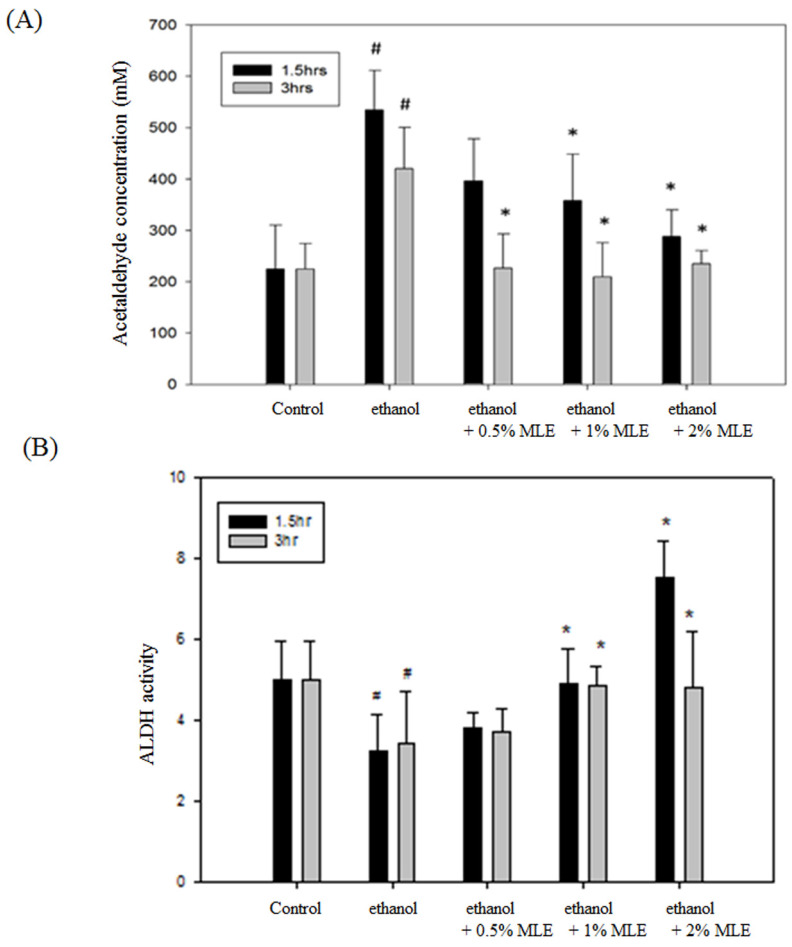
** MLE reduced plasma ethanol and ACE concentration and increased liver ALDH activity in ethanol-induced mice.** (A) ACE concentrations in plasma and (B) liver ALDH activity from ethanol-loaded mice were determined for 1.5 or 3 h after oral administration of 40% ethanol with tested samples. #, p < 0.05 compared with the control group. *: p < 0.05 compared with the ethanol group.

The ACE concentration was detected to determine whether MLE affected the metabolism of ethanol. Fig. 1A shows that the concentration of serum ACE was significantly increased to 420.1 ± 97.4 mM at 3 h post-administration of ethanol. Coadministration of 2% MLE obviously reduced the ACE concentration to 243.8 ± 89.6 mM.

Enzyme activity assay was conducted to verify whether MLE influenced the activities of ALDH. Administration of ethanol for 1.5 and 3 h significantly reduced the activity of ALDH, whereas MLE restored the ALDH activity in the presence of ethanol (Fig. 1B).

### MLE reversed ethanol-induced liver inflammation

Inflammation is one of the major factors that cause liver damages by ethanol. We performed hematoxylin and eosin (H&E) staining and Western blot analysis to determine whether MLE attenuated the ethanol-induced inflammation. H&E staining indicated that ethanol increased leukocyte infiltration in the central vein of liver. MLE treatment repressed the leukocyte infiltration. In addition, ethanol significantly elevated cyclooxygenase-2 (COX-2), interleukin-6 (IL-6), and TNF-α expression levels to 2-, 1.4-, and 1.2-fold compared with control group, respectively. Cotreatment with 1% and 2% MLE reduced the expression levels of COX-2, IL-6, and TNF-α.

### MLE enhanced antioxidant enzyme activities and expression in response to ethanol treatment

Previous reports have indicated that MLE exhibited antioxidant activities. We measured several antioxidant enzyme activities and expression levels to detect whether MLE also increased the antioxidant system in ethanol-treated group. No overt alternation of CAT activity was found. GSH-Px and SOD activities were suppressed by ethanol. Cotreatment with 1% and 2% MLE significantly recovered the activities of GSH-Px and SOD. IHC analysis also demonstrated that expression of SOD in liver was increased in 1% and 2% MLE-treated groups compared with ethanol-treated group.

Previous reports have shown that ethanol induced iNOS and phosphorylation of STAT3 and reduced caveolin-1 expression. We performed IHC analysis to test the effects of MLE on iNOS, p-STAT3, and caveolin-1 expression. The expression of iNOS and p-STAT3 was increased in ethanol-treated groups. MLE reversed the iNOS and p-STAT3 expression.

### MLE reversed the ACE-induced effects of human Hep G2 cells

**Figure 5:**
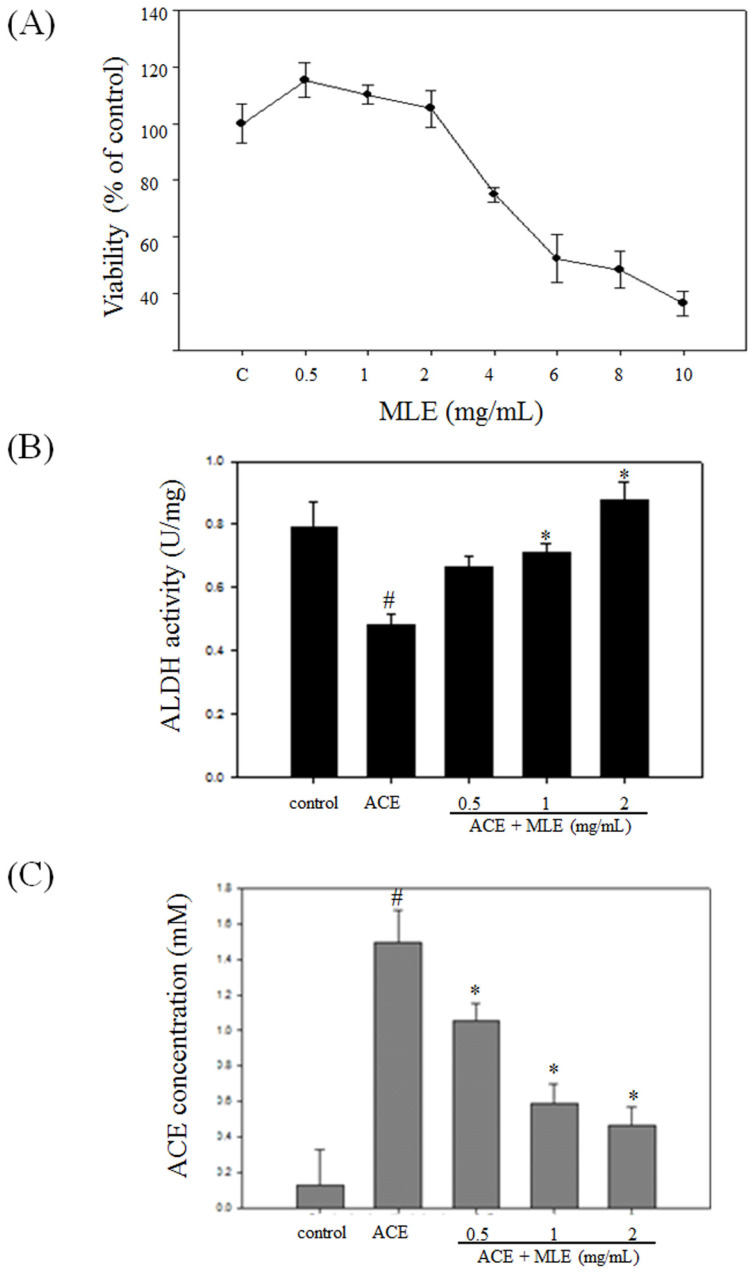
** MLE induced the ALDH activity and decreased the ACE concentration in ACE-induced hepatocyte.** (A) Hep G2 cells were treated with various concentrations of MLE for 24 h. The cell viability was analyzed by MTT assay. The data were mean ± SD from four samples for each group. (B) Hep G2 cells were treated with 175 μM ACE with 0, 0.5, 1, and 2 mg/mL MLE for 3 h. The activity of ALDH and the concentration of ACE were measured. The data were mean ± SD from three independent experiments. #: p < 0.05 compared with the control group. *: p < 0.05 compared with the ACE group. ACE, Acetaldehyde group.

We used human Hep G2 cell to test whether MLE affected the cell viability. The cell viability was significantly decreased in response to 4, 6, 8, and 10 mg/mL MLE, and the IC50 was 7.38 mg/mL (Fig. 5A). We selected 0, 1, and 2 mg/mL MLE for further studies.

Treatment with ACE significantly diminished ALDH activity and increased cellular ACE concentration. MLE dose-dependent treatment increased ALDH activity and decreased ACE concentration compared with ACE-treated group (Figs. 5B and 5C).

### MLE reversed ACE-induced inflammation and oxidative stress

**Figure 6:**
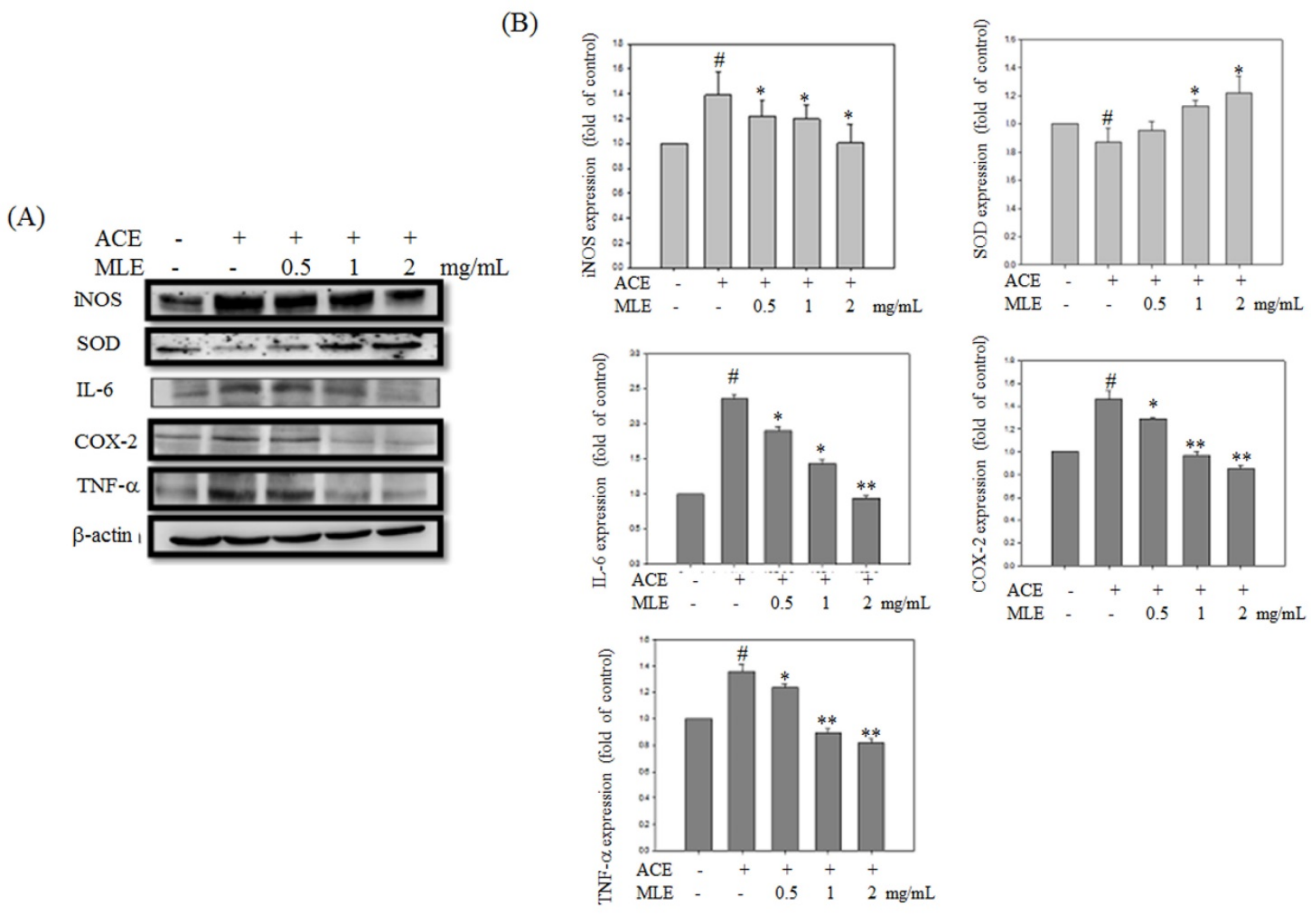
** MLE ameliorated the oxidative stress and inflammation protein expression in ACE-induced hepatocyte.** Hep G2 cells were treated with 175 μM ACE and indicated concentration of MLE for 24 h. The cells were harvested and subjected to Western blot analysis. The data were mean ± SD from three independent experiments. #: p < 0.05 compared with the control group. *: p < 0.05 compared with the ethanol group.

Expression levels of proteins involved in inflammation and oxidative stress were detected to determine whether MLE affected ACE-induced phenomenon. Western blot analysis revealed that inflammation proteins, such as iNOS, IL-6, COX-2, and TNF-α, were significantly increased in ACE exposure groups, whereas SOD expression was inhibited by ethanol. MLE dose-dependent treatment reversed the ACE-induced phenomenon (Fig. 6).

### MLE reduced ethanol-induced apoptosis

**Figure 7:**
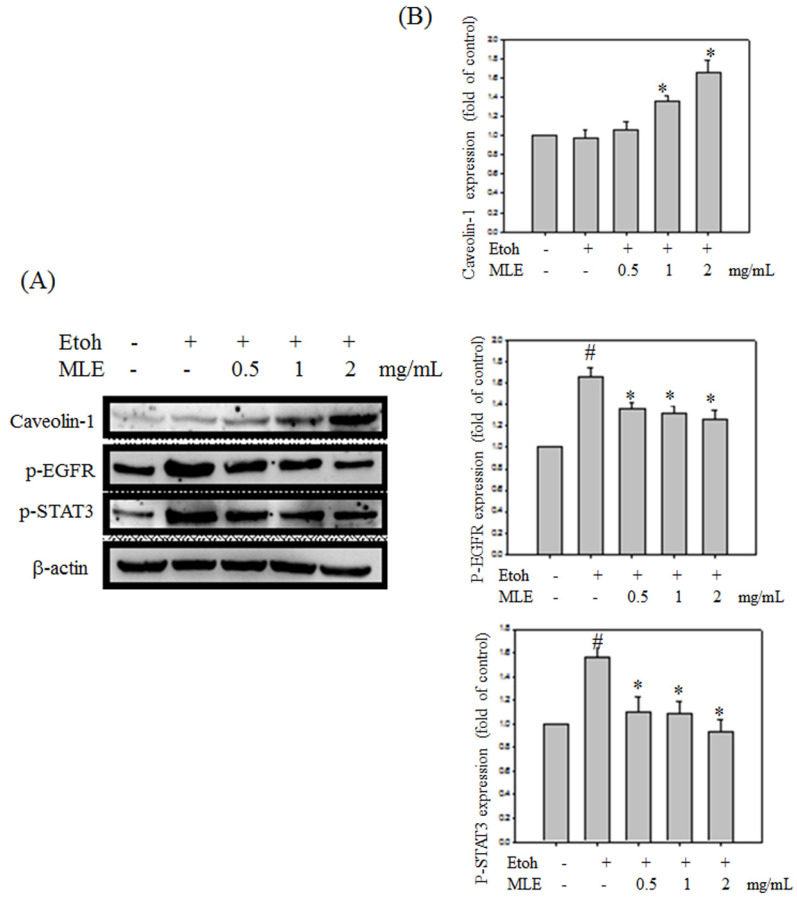
** MLE altered the caveolin-1/pEGFR/p-STAT3 signal in ethanol-induced hepatocyte.** Hep G2 cells were treated with various concentrations of MLE and ethanol for 24 h. The cell lysates were subjected to Western blot analysis. The data were mean ± SD from three independent experiments. #: p < 0.05 compared with the control group. *: p < 0.05 compared with the ethanol group.

We investigated whether MLE regulated caveolin-1 expression and EGFR/STAT3 signal to prevent ethanol-induced cell death in hepatocyte cells. Western blot analysis revealed that ethanol repressed caveolin-1 and enhanced EGFR/STAT3 signals. Cotreatment with MLE significantly reversed the ethanol-induced effects in a dose-dependent manner (Fig. 7).

**Figure 8:**
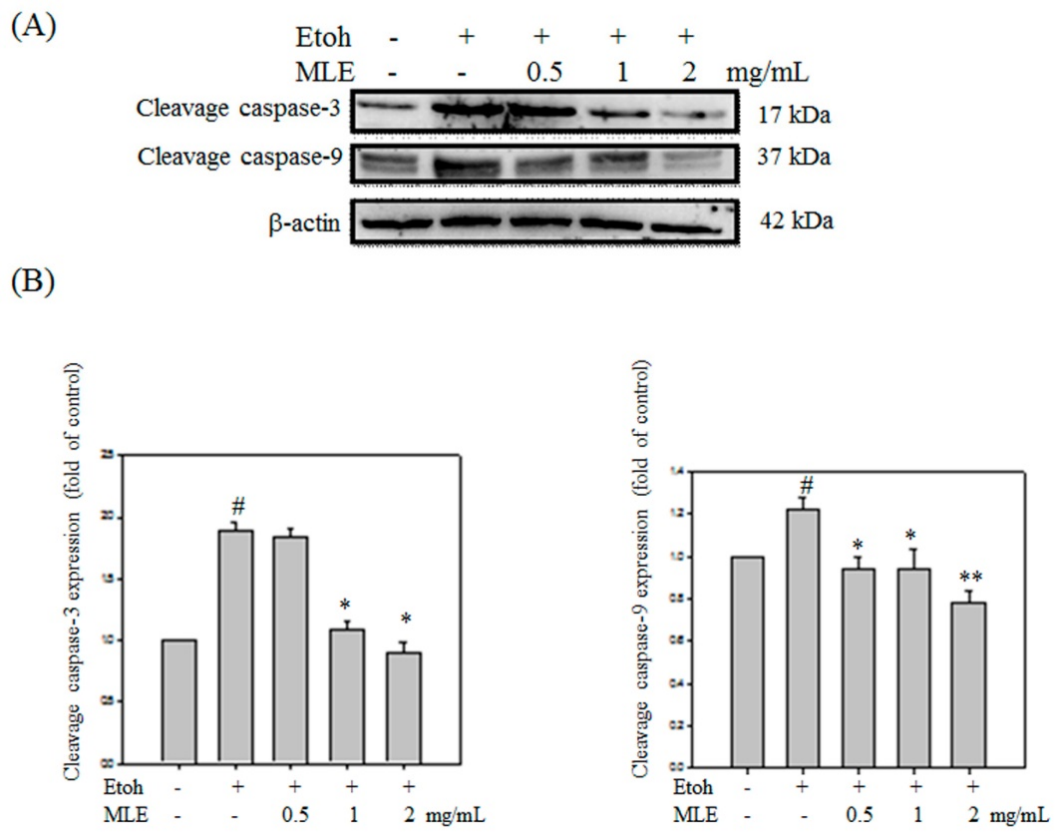
** MLE affected the caspase-3 and -9 cleavage in ethanol-induced hepatocyte.** Hep G2 cells were treated with various concentrations of MLE and ethanol for 24 h. The cell proteins were extracted and subjected to Western blot analysis. The data were mean ± SD from three independent experiments. #: p < 0.05 compared with the control group. *: p < 0.05 compared with the ethanol group. **: p < 0.01 compared with the ethanol group.

Western blot analysis was performed to investigate whether MLE protected ethanol-induced apoptosis in cells. Cleaved caspase-3 and -9 were obviously increased in ethanol exposure groups. Cleaved caspase-3 and -9 was decreased in proportion to MLE concentration (Fig. 8).

## Discussion

Excessive alcoholic assumption may cause liver diseases, such as steatosis, cirrhosis, and hepatocellular carcinoma, which are major health problems 2. Preventing ALD is a current important issue. In this study, we demonstrated that MLE reduced ACE toxicity and reversed alcohol-induced liver apoptosis signals.

Two major steps were involved in alcohol metabolism in liver. First, alcohol was converted into ACE 19. Previous reports have indicated that increased ACE disrupted the mitochondrial functions and led to liver injury 19. ACE adducts also induced immune reaction to promote apoptosis in liver 19. ACE was then metabolized into acetate by ALDH 19. Several reports have indicated that flavonoid elevated the activities of ALDH to attenuate ACE-induced liver injury. Coadministration of Niuchangchih and silymarin significantly increased ALDH activities in response to chronic alcohol treated in male Wistar rats 20. In hippocampal neuronal HT22 cells, rutin, one major compound of MLE, reversed ethanol-induced apoptotic cell death through increased ALDH2 activity 21. In line with these observations, MLE obviously reduced the ACE concentration and increased the ALDH activity in alcohol-treated groups in the present study.

Metabolism of alcohol in liver produced damage-associated danger signals and then triggered pro-inflammatory cytokines, such as TNF-α and IL-6 production 22. These pro-inflammation cytokines recruited immune cells, such as monocyte, macrophage, and neutrophil leukocyte accumulation, in liver and then produced additional pro-inflammation cytokines 23. Reports have shown that elevation of TNF-α and IL-6 was found in serum of ALD patients 24. In the present study, we showed that acute alcohol exposure significantly enhanced the TNF-α and IL-6 levels, whereas MLE significantly attenuated the alcohol-induced inflammation.

ROS plays a critical role in the development of alcohol-induced liver disease. As alcohol was metabolized by ADH and ALDH, cellular ROS level was elevated. ROS caused lipid peroxidation and DNA damage and led to liver injury. Administration of alcohol also influenced the expression levels of several antioxidant enzymes, such as CAT, SOD, and GSH-Px 2, 25. The main function of SOD is to convert superoxide to hydrogen peroxide and water 26. In response to ROS generation, the activity of SOD was increased. However, alcohol intoxication-induced massive ROS repressed the activity of SOD 26. The activity of GSH-Px that converted hydrogen peroxide to water was inhibited because of the depletion of GSH by alcohol 27. Similarly, alcohol also inhibited CAT 27. Reports have shown that extracts from traditional herbs restored alcohol-repressed antioxidant activities. Pinto et al. demonstrated that alcohol diminished SOD activity to 75% of control group, whereas xanthohumol flavonoid extracted from *Humulus lupulus* dose-dependently recovered the alcohol-repressed SOD activities 28. Pretreatment with procyanidins isolated from wild grape seeds clearly reversed the inhibitory effects of alcohol on SOD activity 29. Coadministration of ethanol extract of *Alocasia indica* tuber significantly recovered the SOD activity to normal level in alcohol-intoxicated mice 30. Similarly, extracts from *Portulaca oleracea* and green *Capsicum annuum* obviously enhanced the GSH-Px activity in alcohol-intoxicated rats 31, 32. Previous reports have also shown that MLE elevated the activity of SOD in chronic alcohol administration mice 17. In addition, several compounds from MLE also exhibited liver protection effects against alcohol exposure. Kim et al demonstrated that chlorogenic acid dose-dependent inhibited ethanol-induced lipid accumulation and liver fibrosis 33. In addition, chlorogenic acid also attenuated the ethanol-induced oxidative stress by increased anti-oxidant enzymes activities 33. Rutin exhibited hepatoprotection through elevation of glutathione level, repression of inflammation in HepG2 cells treated with ethanol 34. In line with these observations, we demonstrated that cotreatment with MLE increased the activities of SOD and GSH-Px of acute alcohol-exposed mice in a dose-dependent manner.

Caveolin-1, which is the major component of caveolae, is involved in diverse cellular functions, such as lipid metabolism, regulation of protein activity, and hepatocyte proliferation 35. Caveolin-1 bound to and inhibited the autophosphorylation of EGFR 36. Lo et al. demonstrated that nuclear-translocated EGFR associated with STAT3 and then increased iNOS expression 37. Reports have also demonstrated the important role of caveolin-1 in ALD. Chronic alcohol exposure elevated the expression of caveolin-1 and then inhibited eNOS activity by binding to eNOS 38. Significantly higher iNOS expression was found in depletion of caveolin-1 mice fed with alcohol 39. Caveolin-1 prevented hepatocyte from apoptosis by inhibiting the EGFR/STAT3/iNOS signaling pathway 39. Lee et al. showed that MLE and its major components, namely, chlorogenic acids, elevated caveolin-1 level, then diminished EGFR/STAT3/iNOS pathway, and eventually inhibited hepatocyte apoptosis in chronic alcohol-treated mice 17. In the present study, our results revealed that MLE upregulated caveolin-1 expression, downregulated iNOS, IL-6, and TNF-α, and protected hepatocyte from acute alcohol-induced apoptosis.

**Figure 9:**
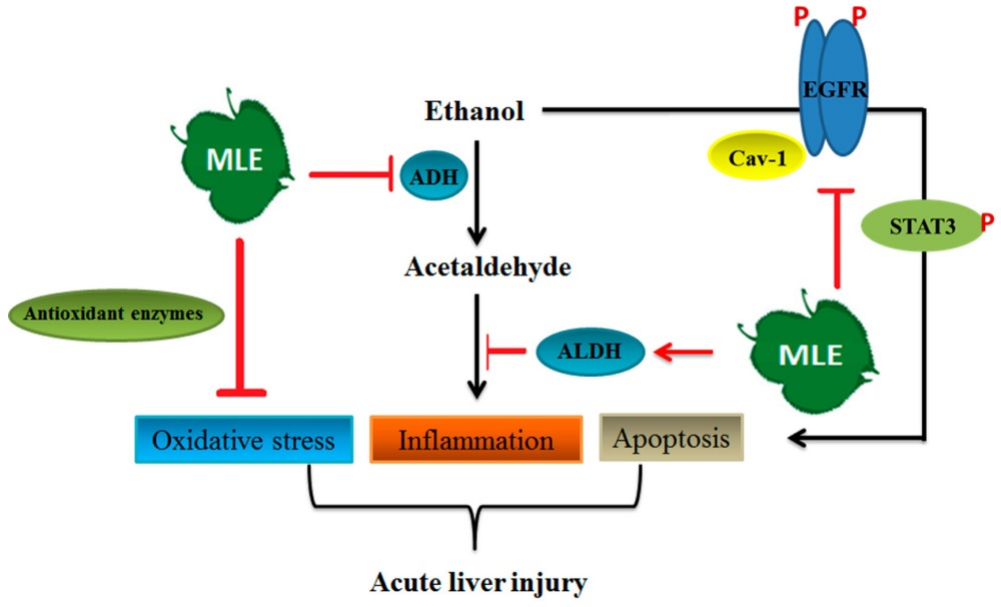
** Summary of the mechanisms of MLE on ethanol-induced effects**. The results showed that MLE reduced serum ACE concentration and promoted ALDH activation. MLE also increased antioxidant enzymes' expression and attenuated inflammation-related proteins' expression. In addition, MLE could reduce apoptosis via caveolin-1/pEGFR/p-STAT3/iNOS pathway.

In conclusion, our results demonstrated that MLE inhibited pro-inflammation factor expression, enhanced antioxidant enzymes' activities, and reversed acute alcohol-induced liver damages. MLE also recovered the ACE-induced toxicity by regulating iNOS, SOD, IL-6, and TNF-α expression in cultured human Hep G2 cells. MLE elevated the caveolin-1 and blocked EGFR/STAT3/iNOS to prevent apoptosis in ethanol-exposed hepatocytes (Fig. 9). Overall, our findings suggested that MLE could be a potential agent for treating ALDs.

## Figures and Tables

**Figure 2:**
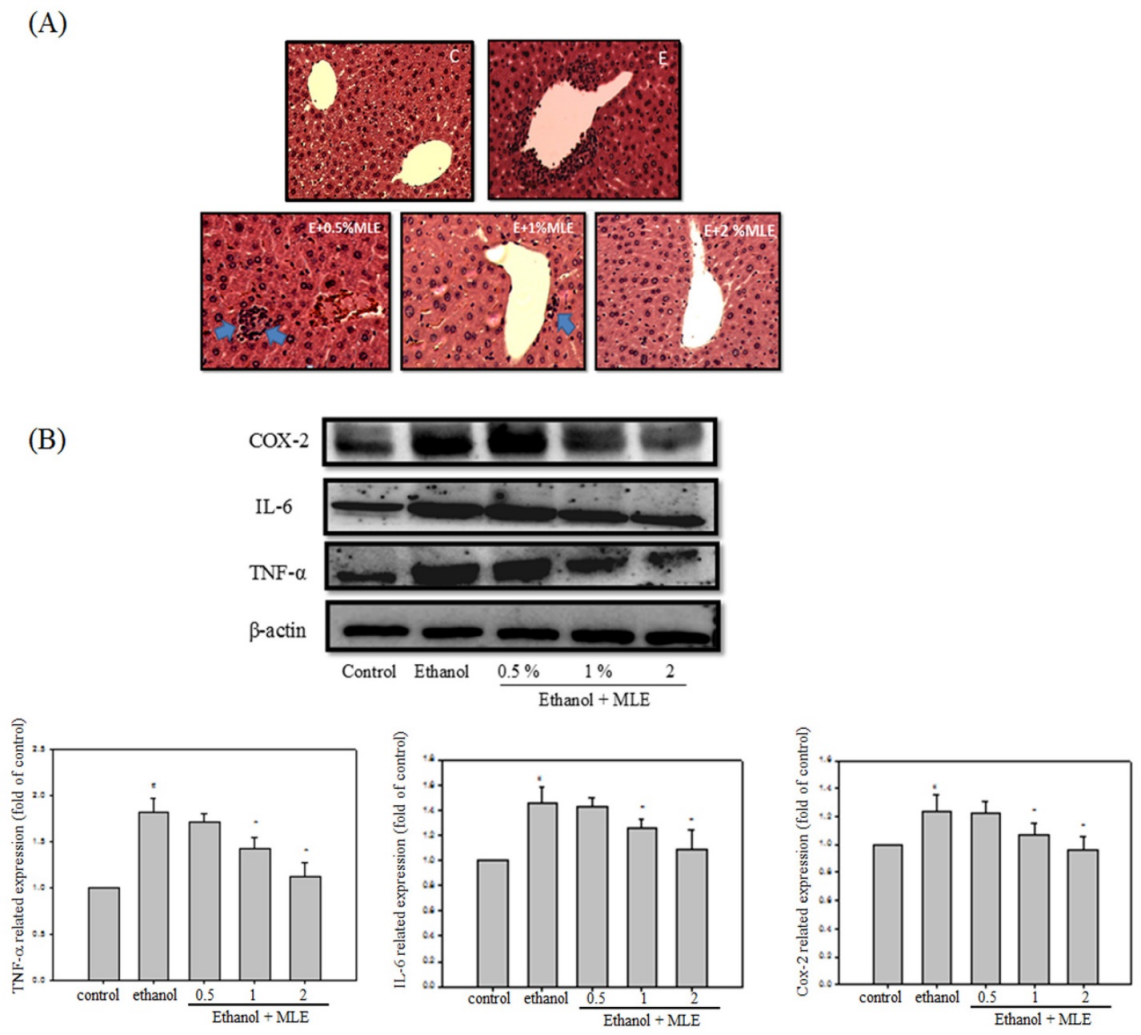
** Liver appearance of acute liver injury from ethanol-induced mice.** Mice were fed with ethanol combined with 0, 0.5%, 1%, or 2 % MLE for 3 h. (A) Paraffin-embedded sections of liver from ethanol-induced mice were stained with hematoxylin and eosin (100×). The results showed that the leukocyte infiltration positive site appeared around the central vein of liver (as indicated by blue arrowhead). (B) The liver tissue was subjected to Western blot analysis. TNF-α, IL-6, and COX-2 were significantly higher in treated ethanol groups. The quantitative data was obtained from three different mice samples. #: p < 0.05 compared with the control group. *: p < 0.05 compared with the ethanol group.

**Figure 3:**
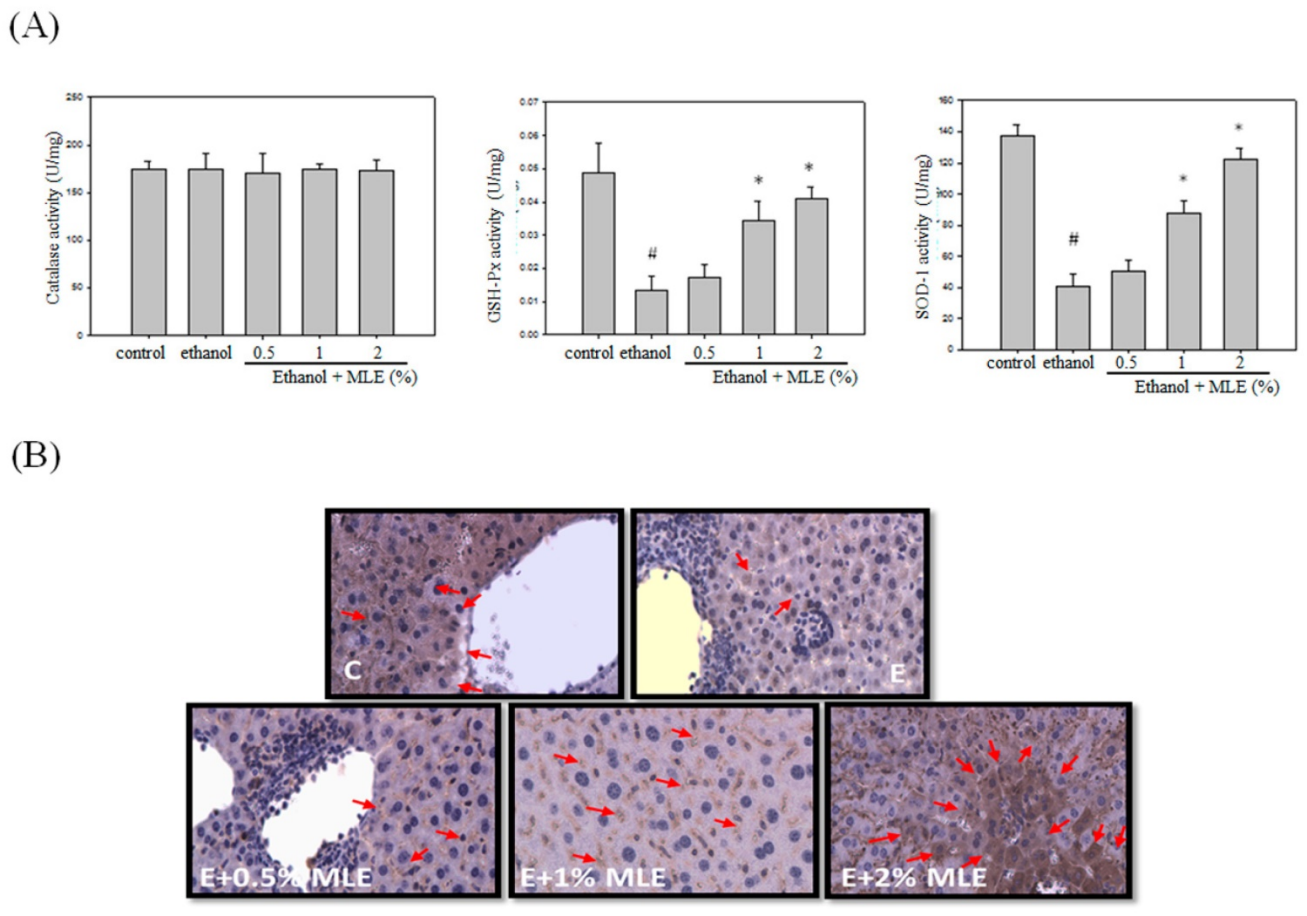
** MLE enhanced the activity and expression of antioxidant enzymes in liver from ethanol-induced mice.** Mice were fed with ethanol combined with 0%, 0.5%, 1%, and 2% MLE for 3 h, (A) Activity of antioxidant enzymes, GSH-Px, and SOD in liver of ethanol-fed mice were determined. #: p < 0.05 compared with the control group. *: p < 0.05 compared with the ethanol group. (B) Expression levels of SOD in mice livers were stained by immunohistochemistry. Representative photomicrographs were magnified 100× and 200×. C, normal group; E, ethanol group. The arrow indicates SOD staining. The red arrow indicated the positive immunohistochemistry signals.

**Figure 4:**
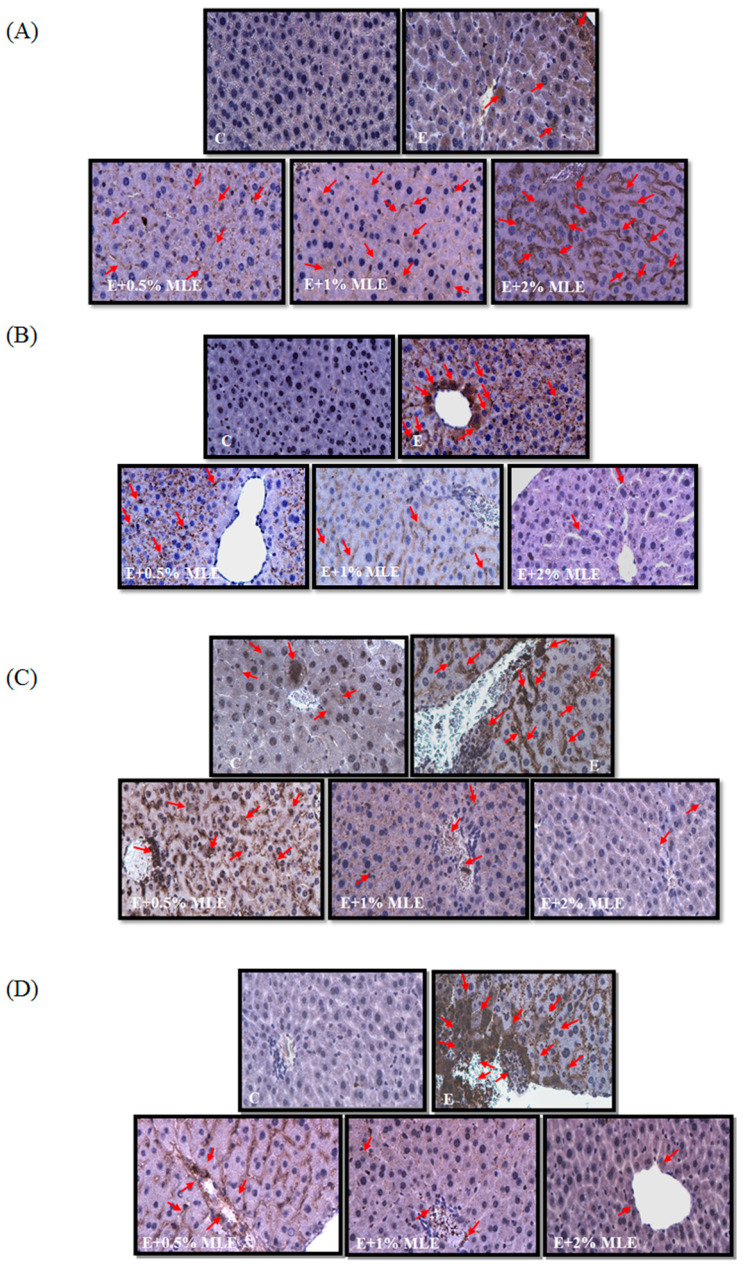
** MLE regulated the expression levels of caveolin-1, p-EGFR, p-STAT3, and iNOS in liver from ethanol-induced mice.** The expression levels of caveolin-1 (A), p-EGFR (B), p-STAT3 (C), and iNOS (D) in mice liver were stained by immunohistochemistry. Representative photomicrographs were magnified 100×. C, normal group; E, ethanol group. The red arrow indicated the positive immunohistochemistry signals.
